# Determinants of COVID-19 Breakthrough Infections and Severity in ChAdOx1 nCoV-19–Vaccinated Priority Groups

**DOI:** 10.4269/ajtmh.22-0172

**Published:** 2022-08-08

**Authors:** Upinder Kaur, Sapna Bala, Bisweswar Ojha, Bhairav Kumar Pathak, Aditi Joshi, Ashish Kumar Yadav, Anup Singh, Sangeeta Kansal, Sankha Shubhra Chakrabarti

**Affiliations:** ^1^ Department of Pharmacology, Institute of Medical Sciences, Banaras Hindu University, Varanasi, Uttar Pradesh, India;; ^2^ Department of Geriatric Medicine, Institute of Medical Sciences, Banaras Hindu University, Varanasi, Uttar Pradesh, India;; ^3^ Institute of Medical Sciences, Banaras Hindu University, Varanasi, Uttar Pradesh, India;; ^4^ Centre for Biostatistics, Institute of Medical Sciences, Banaras Hindu University, Varanasi, Uttar Pradesh, India;; ^5^ Department of Community Medicine, Institute of Medical Sciences, Banaras Hindu University, Varanasi, Uttar Pradesh, India

## Abstract

The current analysis is a part of an ongoing observational study that began in February 2021 in the Sir Sunder Lal Hospital (Varanasi, Uttar Pradesh) in northern India and is expected to continue until June 2022. This analysis aimed to delineate the clinical presentation and risk factors of occurrence and severity of COVID-19 in vaccinated individuals. The study enrolled health-care workers and the elderly receiving the COVID-19 vaccine at one of three centers linked to the study hospital. The participants received the ChAdOx1 nCoV-19 (Oxford-AstraZeneca) vaccine based on the chimpanzee adenovirus platform (manufactured in India by the Serum Institute of India). The adenovirus codes for the spike (S) protein of SARS-CoV-2. Participants were contacted by phone at pre-decided intervals and questioned about the occurrence of COVID-19, clinical presentation, severity, and persistence of symptoms. A logistic regression analysis was performed to predict the risk factors of occurrence and severity of COVID-19. Of the 1,500 participants included in the analysis, 418 developed COVID-19 (27.9%). Fever was the most common symptom (72%), followed by cough (34%) and rhinitis (26%). Cardiovascular involvement was seen in more than 2% of individuals, and 11% had post-COVID-19 complaints. Regression analysis showed 1.6 times greater odds of contracting the disease in females and in those younger than 40 years, 1.4 times greater odds in individuals who were overweight, and 2.9 times greater odds in those receiving only one dose, compared with respective comparators. Individuals receiving two doses at a gap of ≤ 30 days had 6.7 times greater odds of infection than those receiving at a > 60-day interval. There was no association between COVID-19 occurrence in the vaccinees and pre-vaccination history of SARS-CoV-2 infection. Males were at a 3.6 times greater risk, and persons with preexisting lung disease—mainly asthma—had a 5.9 times greater risk of experiencing moderate to severe COVID-19 than comparators. While an extended interval between the two vaccine doses seems to be a better strategy, gender differences and an association of asthma phenotypes with COVID-19 need to be explored.

## INTRODUCTION

COVID-19, caused by SARS-CoV-2, has had a massive impact on health and the economy. Although the disease is mild in most cases, fatalities have been observed in 1% to 3% because of severe respiratory distress, multiorgan failure, and disseminated intravascular coagulopathy.^1^ India experienced a mild form of the pandemic in 2020. Because of the economic impact, the lockdown of 2020 was gradually lifted in early 2021. Newly emerging variants such as the delta variant spread rapidly across the country, and the health sector was overwhelmed by the second wave of the pandemic. COVID-19 peaked in May 2021, with an average daily load of more than 300,000 new cases and a case fatality of ∼1%.^2^ To curtail the impact of the pandemic, various vaccines have been developed at an unprecedented speed and granted emergency use authorization. In India, the primary vaccine administered in the mass rollout is the ChAdOx1-nCoV-19 (Oxford–AstraZeneca) vaccine, based on a recombinant chimpanzee adenovirus coding for the spike protein of SARS-CoV-2. It is manufactured by the Serum Institute of India as COVISHIELD. India’s second most common vaccine is the inactivated SARS-CoV-2 vaccine (BBV152, COVAXIN, Bharat Biotech).^3^^,^^4^ Short-term safety and efficacy of close to 70% have been demonstrated with the ChAdOx1 nCoV-19 in controlled settings. Post-approval, however, a modest reduction in effectiveness has been observed.^5^^,^^6^ We had previously provided the first short-term safety data of COVISHIELD use in the Indian population and, in continuation of the same real-world study, reported the occurrence of COVID-19 in vaccinated priority groups.^4^^,^^7^ Although risk factors of COVID-19 severity are relatively defined in the pre-vaccination period, insights into the patterns and predictors of COVID-19 in the post-vaccination period are equally pertinent, particularly for priority groups such as health-care workers who are at increased risk of disease acquisition. Another factor worth exploring is the effect of dosing interval between the two doses of vaccine on disease occurrence and severity. To contribute to this missing information, the objectives of this study were to provide data on the clinical presentation of COVID-19, atypical presentations of disease and persistent symptoms (long COVID) in vaccine recipients, and, importantly, to identify the determinants of COVID-19 infection and severity in this group.

## MATERIALS AND METHODS

A prospective observational study was initiated in early February 2021 in the Sir Sunder Lal Hospital, a major tertiary university hospital in northern India in Varanasi (Uttar Pradesh), and in two associated government health-care centers. The study was conducted with the primary objective of assessing the short-term and long-term safety profiles of COVID-19 vaccines, with a secondary objective of assessing COVID-19–specific outcomes.^4^ The study is expected to continue until June 2022, with a plan to complete a 1-year follow-up of each vaccinee. The short-term safety data and occurrence rate of COVID-19 in vaccinated individuals have already been published.^4^^,^^7^ Vaccinees were enrolled after obtaining written informed consent.^4^ As per national policy for prioritization of vaccine recipients, the enrolled vaccinees were health-care workers in the initial phase of the study, with the enrollment of elderly individuals in the later stage. Participants were contacted by phone at specific time intervals after vaccination. In our study, they were questioned about the occurrence of COVID-19, clinical features, severity, and persistence of symptoms after 2 months of COVID-19. The exact date of real-time polymerase chain reaction or rapid antigen test positivity was recorded, and so were potential risk factor data. For elderly participants who were not health-care workers, questions were asked in the simple native language and, in case of confusion, the answers were confirmed by caregivers. COVID-19 cases were classified as confirmed and suspected, and severity classification was also performed based on Ministry of Health and Family Welfare guidance and as described previously.^7^ Any person with laboratory-confirmed SARS‐CoV‐2 infection, regardless of clinical signs or symptoms, was considered a confirmed case. COVID‐19 suspected cases were those with a suggestive clinical pattern of symptoms and exposure to cases of COVID‐19 within the past 14 days, but without laboratory confirmation. To simplify analyses, both confirmed and suspected cases are included in group A, with group B including the remaining cases. Similarly, for determining risk factors of severe disease, individuals with asymptomatic or mild disease were incorporated into one group. They were compared with individuals who developed moderate or severe COVID-19. Both groups had similar vaccination timings (mid-January to early March 2021). Potential risk factors analyzed for both occurrence and severity of COVID-19 were demographic variables, comorbidities, use of renin–angiotensin–aldosterone system (RAAS) blockers, doses of vaccine received, and the interval between the two doses. Post-COVID-19 symptoms, if any, at 2 months of follow-up were also assessed.

### Statistical analysis.

The sample size calculated for this study was 1,650, the details of which have been reported previously.^4^ Bivariate analysis was performed for risk factor determination using the χ^2^ test for dichotomous variables, and categorized quantitative variables such as body mass index and age. Variables with *P* < 0.05 and those deemed clinically relevant were incorporated into the final regression model. Corroboration was done with the multivariable fractional polynomial method (STATA 16; StataCorp LLC; College Station, TX). To determine the effect of the dosing interval of vaccines on COVID-19 occurrence and severity, regression analysis was done separately after excluding those who received only one dose and those who developed COVID-19 between the two doses of vaccine. In the case of a significant correlation (*r *> 0.7) between independent variables, the variance inflation factor was used to detect multicollinearity (cutoff, 2.5).

## RESULTS

**Figure 1.**
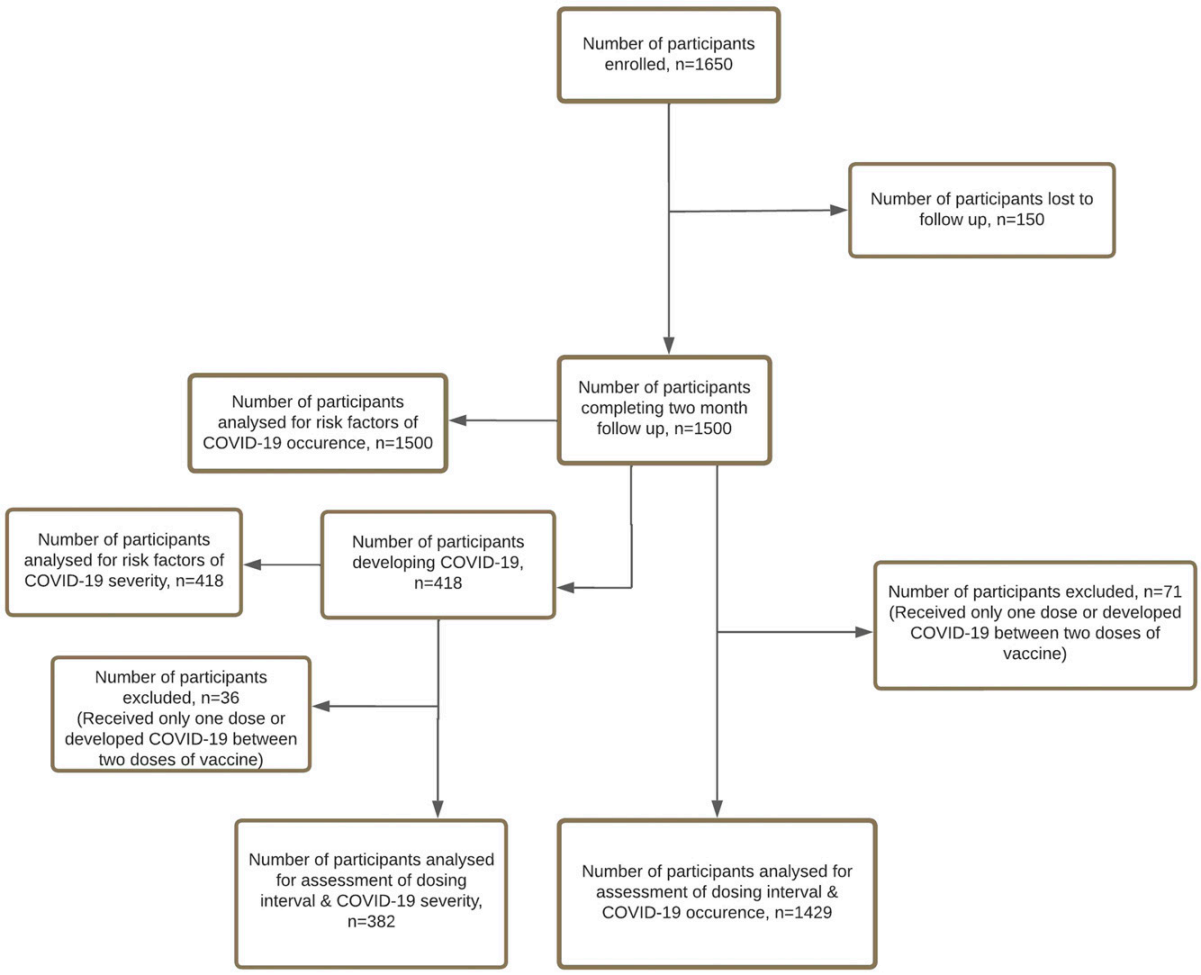
STROBE flow diagram of the study with participants analyzed at each step.

A total of 1,650 individuals were enrolled in the study, of whom 150 were lost to follow-up at the time of telephone contact (i.e., at least 2 months since the last dose of vaccine). Thus, a total of 1,500 vaccinated individuals completing at least 2 months of follow-up could be contacted successfully. Of these 1,500, 418 individuals were diagnosed as cases of COVID-19 (confirmed or suspected, group A) based on the telephone information and laboratory reports shared with the authors by the enrolled individuals.^7^ Figure 1 shows the flow diagram of the study. Supplemental Table S1 shows the demographic parameters and comorbidities in the two groups, detailed clinical features, treatment received, and post-COVID-19 complaints (group A). It also shows the bivariate analysis to determine risk factors of COVID-19 occurrence. Table 1 details the results of the binary logistic regression analysis (*N* = 1,500) to confirm these risk factors. A separate regression analysis to determine the effect of the dosing interval of the vaccine on disease occurrence (*N* = 1,429) is also shown in Table 1. Younger individuals (< 40 years), females, overweight individuals, and those who received only one dose of vaccine had 1.6, 1.6, 1.4, and 2.9 times greater odds, respectively, of contracting COVID-19, with statistical significance. The odds of contracting COVID-19 was 6.7 times in those receiving the two doses at ≤ 30 days compared with those who received the second dose at a gap of > 60 days (*P* = 0.01). Supplemental Table S2 describes the results of the bivariate analysis performed on individuals developing COVID-19 (*n* = 418) to identify risk factors of moderate to severe disease. Table 2 describes the logistic regression analysis of the risk factors for moderate to severe forms of COVID-19. To determine the effect of dosing interval on the severity of disease, logistic regression was performed on a smaller set (*n* = 382) of individuals (Table 2). Males, recipients of only one dose, and those with preexisting lung disease had 2.9, 2.9, and 5.9 times greater odds, respectively, of developing a moderate to severe form of the disease than respective comparators (Table 2). The risk association was statistically significant for each. The association of RAAS blockers with the severity of illness was nullified in the regression analysis (Supplemental Table S3).

**Table 1:** Regression analysis to determine tentative risk factors for occurrence of COVID-19 in vaccinated priority groups

Regression analysis of 1,500 participants			Regression analysis 1,429 participants		
Tentative risk factors	OR (CI)	*P* value	Tentative risk factors*	OR (CI)	*P* value
Age, y					
< 40	1.6 (1.3–2.1)	< 0 .001	< 40	1.7 (1.3–2.2)	< 0.001
≥ 40 (ref.)	–	–	≥ 40 (ref.)	–	–
Gender					
Female	1.6 (1.3–2.1)	< 0.001	Female	1.7 (1.3–2.2)	< 0.001
Male (ref.)	–	–	Male (ref.)	–	–
Body mass index, kg/m^2^					
≥ 25	1.4 (1.1–1.8)	< 0 .01	≥ 25	1.44 (1.13–1.85)	0.003
< 25 (ref.)	–	–	< 25 (ref.)	–	–
No. of vaccine doses			Dosing interval, d		
1	2.9 (1.8–4.7)	< 0.001	≤ 30	6.7 (1.6–28.6)	0.01
2 (ref.)	–	–	> 60 (ref.)		
	–	–	≤ 30	1.6 (1.13–2.32)	0.008
	–	–	31–60 (ref.)		
	–	–	31–60	4.1 (0.95–18)	0.056
	–	–	> 60 (ref.)		
Hypothyroidism					
Yes	1.18 (0.66–2.1)	0.58	Yes	1.05 (0.56−1.9)	0.86
No (ref.)			No (ref.)	–	–

OR = odds ratio.

* After excluding those who received only one dose and those who developed COVID-19 between the two doses.

**Table 2:** Logistic regression analysis of the risk factors of moderate to severe COVID-19 in vaccinated individuals developing COVID-19

Regression analysis of 1,500 participants			Regression analysis 1,429 participants		
Tentative risk factors (*n* = 418)	OR (CI)	*P *value	Tentative risk factors (*n* = 382)*	OR (CI)	*P* value
Age, y					
≥ 40	1.14 (0.85–2.4)	0.73	≥ 40	1.35 (0.6–3)	0.46
< 40 (ref.)	–	–	< 40 (ref.)	–	–
Gender					
Male	2.9 (1.3–6.2)	0.008	Male	3.6 (1.4–9.4)	0.007
Female (ref.)	–	–	Female (ref.)	–	–
Body mass index, kg/m^2^					
≥ 25	1.4 (0.7–2.7)	0.31	≥ 25	1.73 (0.8–3.7)	0.14
< 25 (ref.)	–	–	< 25 (ref.)	–	–
Diabetes mellitus					
Yes	1.35 (0.5–3.8)	0.56	Yes	1.3 (0.4–4.3)	0.65
No (ref.)	–	–	No (ref.)	–	–
Hypertension					
Yes	1.3 (0.5–3.6)	0.62	Yes	1.1 (0.32–3.7)	0.89
No (ref.)	–	–	No (ref.)	–	–
Heart disease					
Yes	1.82 (0.26–12.5)	0.54	Yes	NA†	NA†
No (ref.)	–	–	No (ref.)	–	–
Lung disease					
Yes	3.3 (0.83–12.5)	0.08	Yes	5.9 (1.3–27.8)	0.024
No (ref.)	–	–	No (ref.)	–	–
No. of vaccine doses			Dosing interval, d		
1	2.9 (1.2–7.2)	0.016	≤ 30	5 (0.64–33.3)	0.13
2 (ref.)	–	–	> 30 (ref.)	–	–

NA = not assessed; OR = odds ratio; ref. = reference.

* After excluding those who received only one dose and those who developed COVID-19 between the two doses.

† Small sample size; hence, could not be assessed.

## DISCUSSION

Compared with controlled settings, a modest reduction has been observed in vaccine effectiveness in real-world situations, with protection rates varying from 24% to 65%.^5^^,^^6^ We analyzed the spectrum of symptoms of COVID-19 in vaccinated individuals, and the risk factors of breakthrough infections and their severity. The most common symptom was fever (72%), followed by cough and rhinitis. Approximately 5% of individuals with laboratory-confirmed COVID-19 were asymptomatic. Cardiovascular involvement—mainly, hypertension—was observed in 2.6% of individuals, and ocular symptoms in > 2%, emphasizing the need to be vigilant for atypical clinical features in the face of emerging variants of SARS-CoV-2. Approximately 11% had some post-COVID-19 complaint at the 2-month follow up, the most common being generalized weakness. This attains special significance in future deliberations of whether vaccines have a role in preventing long COVID.

On logistic regression analysis, the occurrence of COVID-19 was more common in females (odds ratio [OR], 1.6), in the younger age group (OR, 1.6), in individuals who were overweight (OR, 1.4), and in those who received only one dose (OR, 2.9), with statistical significance. High body mass index, previously, has been associated with severe COVID-19, but we could not find any reported link with occurrence.^8^^,^^9^ Although males and females are believed to have similar susceptibility to disease acquisition, some studies have shown a female preponderance.^10^ In certain regions of Peru, the incidence rate of COVID-19 was shown to be greater in females.^11^ No difference in the occurrence of COVID-19 was seen with respect to other variables such as pre-vaccination history of COVID-19, comorbidities, and use of RAAS blockers. The absence of an association between COVID-19 occurrence and pre-vaccination history of SARS-CoV-2 infection in vaccinated individuals may imply that the claims of the boosting effect of vaccines on naturally acquired immunity need further scrutiny. Dosing interval was observed to be a significant influencer of the occurrence of COVID-19. A 6.7 times and 1.6 times greater risk of infection was observed in individuals receiving two doses of vaccine at ≤ 30-day intervals compared with those receiving at > 60 days and 31 and 60 days, respectively. The corresponding OR was 4.1 in those receiving the second dose at 31 to 60 days compared with at > 60 days (*P* = 0.056). These observations have been largely unexplored in the real world but are in line with exploratory findings in controlled settings.^12^

With respect to the severity of COVID-19, a statistically significant association was seen with gender, preexisting lung disease, use of RAAS blockers, and number of vaccine doses. Except for use of RAAS blockers, the association of the remaining three remained statistically significant on logistic regression analysis. The association of RAAS blockers with disease severity has been refuted in some large meta-analyses.^13^^,^^14^ Males and those receiving only one dose had close to three times greater odds of developing moderate to severe disease. With marginal statistical significance (*P* = 0.08), individuals with preexisting lung disease had more than three times the risk of developing moderate to severe COVID-19. These risk associations were enhanced in the regression analysis confined to individuals receiving two doses of vaccine (*n* = 382), with an OR of 3.6 for male gender and an OR of 5.9 for lung disease. Males have been reported to be at greater risk of severe forms of COVID-19 in several studies in the pre-vaccination period.^8^^,^^9^ Increased severity in men might be explained by androgen-facilitated SARS-CoV-2 entry. Testosterone can upregulate the expression of angiotensin-converting enzyme 2 and possibly transmembrane serine protease 2.^15^ Testosterone is also known to blunt the protective functions of neutrophils, macrophages, and dendritic cells.^16^ Asthma was the predominant lung disease in our vaccinated cohort. Evidence surrounding the association of asthma with COVID-19 is conflicting. Although the CDC suggests moderate to severe asthma to be a potential risk factor for severe COVID-19, no significant association was claimed in a study by Muñoz et al.^17^ Severity of COVID-19 may also be governed by the T-helper cell-phenotype of asthma.^17^^,^^18^ Unlike with incidence, dosing interval was not a statistically significant predictor of severity of disease.

Our study was designed for vaccinated individuals only, and comments on vaccine effectiveness cannot be made. Being a telephone survey-based study, the possibility of recall bias may exist. However, the participants were primarily health-care workers, and the data provided can be considered mostly reliable. Furthermore, we feel recall bias would not be significant with monthly monitoring. Telephone survey-based methods may also have limitations with respect to in-person interviews and objective evaluation. COVID-19 symptoms, history of contact with COVID-19 cases, and findings of laboratory tests for SARS-CoV-2 were discussed on the phone. This was done in accordance with the original study protocol and prevalent social-distancing norms. SARS-CoV-2 anti-spike protein antibodies could not be measured because of funding issues and the study design. Thus, asymptomatic SARS-CoV-2 infections might have been missed. Furthermore, variant-specific data were not obtained, but separate reports from the institute during the study period reported a dominance of delta (B.1.617.2), which drove the second wave in India.^7^ Precise estimates of exposure were not available for each vaccinee, except details provided by participants, but considering they were mostly health-care workers on active duty during the rampant spread of the delta variant in the second wave, exposure levels may be considered similar in both groups A and B. Mostly, older age, presence of cardiovascular disease, obesity, and diabetes have been related to poorer COVID-19 outcomes. Because our study primarily involved younger health-care workers, the prevalence of these comorbidities in our sample was low and hence not reflective of general population demographics. The study findings, therefore, may not be generalized to other populations. Future studies with better characterization of elderly individuals and people with comorbidities are needed to generate risk group-specific findings.

To our knowledge, no study so far has determined the risk factors of both occurrence and severity of breakthrough infections after COVID-19 vaccination, particularly in vaccinated priority groups. One recent publication predicted the determinants of breakthrough infections in the adult population of Belgium. The study had a similar prospective cohort design as ours, but retrieved data from health and social sector registries. Vaccination with ChAdOx1 nCov-19 was associated with a nearly 1.7 times greater risk of breakthrough infections compared with messenger RNA-based vaccines. In concordance with our findings, the risk of breakthrough infections was greater in younger populations.^19^ Risk factors for severity of breakthrough infections were, however, not determined, and neither were the findings specific to high-risk groups. Identification of determinants of COVID-19 outcomes specific to health-care workers and the elderly is essential to govern future vaccination policies for these priority groups. Our study is also distinctive in exploring the real-world effect of the timing of doses of the ChAdOx1 vaccine on COVID-19 occurrence and severity, from a prospective design.

Among the vaccinated, younger individuals, women, and overweight individuals have a greater risk of contracting COVID-19. Men and those with lung diseases such as asthma were seen to have a greater risk of contracting moderate to severe COVID-19. No association was observed between the occurrence and severity of COVID-19 and pre-vaccination SARS-CoV-2 infection. The claims of the boosting effect of vaccination on naturally acquired immunity need further scrutiny. A longer dosing interval was found to protect against disease, emphasizing the need for planning the optimal timing of the dosing schedule. Further focused studies are needed to investigate gender differences and the risk association of COVID-19 with asthma phenotypes.

## Supplemental files


Supplemental materials
