# Supplementary material for: Determinants of COVID-19 Breakthrough Infections and Severity in ChAdOx1 nCoV-19–Vaccinated Priority Groups
Source: Am J Trop Med Hyg. 2022 Aug 8;107(4):850–5. doi: 10.4269/ajtmh.22-0172 (PMC9651529; doi:10.4269/ajtmh.22-0172)
Supplement: Supplementary file 1 [file tpmd220172.SD1.pdf]

Supplementary Table 1. Demographic parameters, comorbidities, clinical features

	Group A n=418	Group B n=1082	P value
Median age (years) (Q1, Q3)	34 (28,43)	35 (29,48)	0.048
<40 years, n (%)	286 (68.4)	640 (59)	**0.001**
≥40 years, n (%)	132 (31.6)	442 (41)	
Males/Females	258/160	770/312	**<0.001**
BMI (Kg/m^2) Mean ± SD	25 ±3.45	24.76 ± 3.63	0.31
BMI ≥ 25, n (%)	205 (49)	473 (43.7)	0.06
Diabetes mellitus, n (%)	40 (9.6)	102 (9.4)	0.93
Hypertension, n (%)	45 (10.8)	125 (11.6)	0.66
On RAAS blockers[#]	29 (6.9)	75 (6.9)	0.99
Heart disease, n (%)	6 (1.4)	17 (1.6)	0.85
Lung disease, n (%)	13 (3.1)	29 (2.7)	0.65
Hypothyroidism, n (%)	20 (4.8)	34 (3.1)	0.13
Pre-vaccination history of COVID-19, n (%)	51 (12.2)	153 (14.1)	0.33
Two dose recipients, n (%)	382 (91.4) *	1047 (96.8)	**<0.001**
Only one dose recipient	36 (8.6)	35 (3.2)	
Number of symptoms (range)	1-9	NA	
Median number of symptoms (Q1, Q3)	3 (2-4)		
TTR range in days	1-75		
Median TTR, Days (Q1, Q3)	7 (4,13)		

Clinical Features (n=418)	n (%)
Fever	300 (71.8)
Cough	142 (34)
Rhinitis	108 (25.8)
Sore throat	99 (23.7)
Weakness	96 (23)
Only weakness[¶]	1 (0.2)
Malaise	21 (5)
Body ache	82 (19.6)
Only body ache[¶]	1 (0.2)
Anosmia	58 (13.9)
Headache	57 (13.6)
Only headache[¶]	1 (0.2)
Dyspnoea	34 (8.1)
Myalgia	31 (7.4)
Only myalgia[¶]	1 (0.2)
Diarrhoea	24 (5.7)
Loss of taste	21 (5)
Tachycardia	17 (4.1)
Chest discomfort	14 (3.3)
Anorexia	5 (1.2)
Backache	7 (1.7)
Only backache[¶]	1 (0.2)
Altered taste	6 (1.4)
**Blood pressure changes**	11 (2.6)
Increase in BP	9 (2.1)
Low BP	1 (0.2)
Fluctuating BP	1 (0.2)
**Eye symptoms**	9 (2.1)
Burning sensation in eyes	3 (0.7)
Watery eyes	2 (0.5)
Redness in eyes	2 (0.5)
Pain in eyes	1 (0.2)
Conjunctivitis (diagnosed)	1 (0.2)
**Others**	
Nausea/vomiting	8 (1.9)
Abdominal discomfort	7 (1.7)
Anxiety/uneasiness	4 (0.9)
Weight loss	4 (0.9)
Rash	3 (0.7)
Only rash[¶]	1 (0.2)
Tachypnoea	3 (0.7)
Dry mouth	3 (0.7)
Dry throat	4 (0.9)

Treatment received	
**Antimicrobials**	
Doxycycline	209
Ivermectin	202
Azithromycin	185
Beta-lactams	20
Favipiravir	15
Remdesivir	11
Metronidazole	4
DEC	1
Linezolid	1
**Supportive**	
**Anticoagulants**	
LMWH	9
Oral factor Xa inhibitors	5
**Antiplatelets**	6
**Others**:	
Antihistaminic	42
Antiarrhythmic	1
Ayurvedic	10
Only Ayurvedic	3
Baclofen	1
Aripiprazole	1

Post COVID-19 complaints in n=45 (10.7%)**

Weakness	20 (44.4[##])
Cough	8 (17.8)
Dyspnoea	6 (13.3)
Backache	3 (6.7)
Bodyache	3 (6.7)
Chest discomfort	3 (6.7)
Headache	3 (6.7)
Anosmia	1 (2.2)
Hyposmia	1 (2.2)
Diarrhoea	1 (2.2)
Difficulty in recalling names	1 (2.2)
Dryness of nose	1 (2.2)
Eye symptoms	1 (2.2)
Fever	1 (2.2)
Hypertension	1 (2.2)
Itching in hands	1 (2.2)

				Dizziness	3 (0.7)	Joint pain	1 (2.2)
				Insomnia	3 (0.7)	Loss of taste	1 (2.2)
				Joint pain	2 (0.5)	Rheumatoid arthritis aggravated	1 (2.2)
				Neck pain	2 (0.5)		
				Increased thirst	2 (0.5)	Shivering	1 (2.2)
				Shivering	2 (0.5)	Sleep disturbance	1 (2.2)
				Palpitations	2 (0.5)	Sore throat	1 (2.2)
				Constipation	1 (0.2)	Syncope	1 (2.2)
				Bloating	1 (0.2)	Tachycardia	1 (2.2)
				Increased appetite	1 (0.2)	Thrombocytopenia	1 (2.2)
				Increased craving for sweet food	1 (0.2)	Tremors on standing	1 (2.2)
				Lymph node swelling	1 (0.2)		
				Burning sensation in urine	1 (0.2)		
				Hiccups	1 (0.2)		
				Jaundice	1 (0.2)		
				Syncope	1 (0.2)		
				Drowsiness	1 (0.2)		
				Atrial fibrillation (in patient of CHF)	1 (0.2)		
				Difficulty in talking	1 (0.2)		
				Oral ulcer	1 (0.2)		
				Itching	1 (0.2)		
				Wheeze	1 (0.2)		
				Atypical behaviour	1 (0.2)		
				Tingling sensation in body	1 (0.2)		
				Only tingling sensation[¶]	1 (0.2)		

[* Of 418 in Group A, 388 received two doses, six of them developed COVID-19 before receiving the second dose

[#]Included ACEI or ARBs or both

[¶]RT-PCR confirmed case

**Out of all vaccinees developing COVID-19; [##]All individual post-COVID-19 complaint percentages are out of individuals developing post-COVID-19 complaints, n=45

Abbreviations: BMI: body mass index, COVID-19: Corona virus disease-2019, DEC: diethylcarbamazine, LMWH: low molecular weight heparin, RAAS: renin angiotensin aldosterone system]

Supplementary Table 2: Bivariate analysis to determine risk factors of severity of disease in individuals developing COVID-19 (n=418)

	Asymptomatic-mild COVID-19, n=371	Moderate-severe COVID-19, n=47	P value
**Median age (years, (Q1, Q3))**	33 (28,42)	37 (28.5,49.5)	0.055
**Age-categories**			
**< 40 years**	258 (69.5)	28 (59.6)	0.16
**≥ 40 years**	113 (30.5)	19 (40.4)	
**Sex (Female/Male)**	151/220	9/38	**0.004**
**Co-morbidities**			
**BMI (Kg/m^2) categories**			
**≥25**	178 (48)	27 (57.4)	0.22
**<25**	193 (52)	20 (42.6)	
**DM, n (%)**	32 (8.6)	8 (17)	0.06
**HTN, n (%)**	37 (10)	8 (17)	0.14
**RAAS blockers, n (%)**	22 (5.9)	7 (14.9)	**0.023**
**Heart disease, n (%)**	4 (1.1)	2 (4.3)	0.084
**Lung disease, n (%)**	9 (2.4)	4 (8.5)	**0.024**
**Hypothyroidism, n (%)**	18 (4.9)	2 (4.3)	0.86
**Two dose recipients, n (%)**	345 (93)	37 (78.7)	**0.001**
**Pre-vaccination history of COVID-19, n (%)**	43 (11.6)	8 (17)	0.28

[BMI: body mass index, COVID-19: Corona virus disease-2019, DM: diabetes mellitus, HTN: hypertension, RAAS: renin angiotensin aldosterone system]

Supplementary Table 3: **Binomial logistic regression analysis showing association between severity of COVID-19 and potential risk factors including use of RAAS blockers**

**Tentative risk factors (n=418)**	**OR (CI)**	**P value**
**Sex**		
Male	2.9 (1.36-6.6)	**0.006**
Female (reference)		
**Age (years)**		
≥40	1.1 (0.5-2.3)	0.86
<40 (reference)		
**BMI (Kg/m²)**		
≥25	1.4 (0.74-2.7)	0.3
<25 (reference)		
**Diabetes mellitus**		
Yes	1.25 (0.45-3.6)	0.66
No (reference)		
**Heart disease**		
Yes	1.6 (0.24-11.5)	0.61
No (reference)		
**Lung disease**		
Yes	3.3 (0.87-12.3)	0.08
No (reference)		
**Use of RAAS blockers#**		
Yes	2.1 (0.7-6.2)	0.18
No (reference)		
**Number of doses of vaccine**		
1	2.8 (1.14-6.8)	**0.025**
2 (reference)		

[#Significant correlation (r>0.7) was seen between hypertension and use of RAAS blockers with variance inflation factor value of >2.5. To exclude multi-collinearity, hypertension was excluded from this analysis.

BMI: body mass index, CI: confidence interval, RAAS: renin angiotensin aldosterone system, OR: Odds ratio]
